# MicroRNA-1301-Mediated RanGAP1 Downregulation Induces BCR-ABL Nuclear Entrapment to Enhance Imatinib Efficacy in Chronic Myeloid Leukemia Cells

**DOI:** 10.1371/journal.pone.0156260

**Published:** 2016-05-26

**Authors:** Tsung-Yao Lin, Ku-Chung Chen, Hsing-Jin Eugene Liu, Ann-Jeng Liu, Kun-Li Wang, Chwen-Ming Shih

**Affiliations:** 1 Graduate Institute of Medical Sciences, College of Medicine, Taipei Medical University, Taipei, Taiwan; 2 Department of Biochemistry and Molecular Cell Biology, College of Medicine, Taipei Medical University, Taipei, Taiwan; 3 Division of Hematology Oncology, Department of Medicine, Wan Fang Hospital, Taipei Medical University, Taipei, Taiwan; 4 Department of Neurosurgery, Taipei City Hospital Ren-Ai Branch, Taipei, Taiwan; 5 Institute of Chemical Engineering, National Taipei University of Technology, Taipei, Taiwan; Università degli Studi di Firenze, ITALY

## Abstract

Chronic myeloid leukemia (CML) is a myeloproliferative disease. Imatinib (IM), the first line treatment for CML, is excessively expensive and induces various side effects in CML patients. Therefore, it is essential to investigate a new strategy for improving CML therapy. Our immunoblot data revealed that RanGTPase activating protein 1 (RanGAP1) protein levels increased by approximately 30-fold in K562 cells compared with those in normal cells. RanGAP1 is one of the important components of RanGTPase system, which regulates the export of nuclear protein. However, whether RanGAP1 level variation influences BCR-ABL nuclear export is still unknown. In this report, using shRNA to downregulate RanGAP1 expression level augmented K562 cell apoptosis by approximately 40% after treatment with 250 nM IM. Immunofluorescence assay also indicated that three-fold of nuclear BCR-ABL was detected. These data suggest that BCR-ABL nuclear entrapment induced by RanGAP1 downregulation can be used to improve IM efficacy. Moreover, our qRT-PCR data indicated a trend of inverse correlation between the *RanGAP1* and microRNA (miR)-1301 levels in CML patients. MiR-1301, targeting the *RanGAP1* 3′ untranslated region, decreased by approximately 100-fold in K562 cells compared with that in normal cells. RanGAP1 downregulation by miR-1301 transfection impairs BCR-ABL nuclear export to increase approximately 60% of cell death after treatment of 250 nM IM. This result was almost the same as treatment with 1000 nM IM alone. Furthermore, immunofluorescence assay demonstrated that Tyr-99 of nuclear P73 was phosphorylated accompanied with nuclear entrapment of BCR-ABL after transfection with RanGAP1 shRNA or miR-1301 in IM-treated K562 cells. Altogether, we demonstrated that RanGAP1 downregulation can mediate BCR-ABL nuclear entrapment to activate P73-dependent apoptosis pathway which is a novel strategy for improving current IM treatment for CML.

## Introduction

Imatinib (IM) is used as a first line drug for chronic myeloid leukemia (CML) therapy. Currently, CML drugs including IM and second generation drugs are very expensive, and this expense may reduce the opportunity for CML patients to receive appropriate therapy [1]. The annual cost of IM therapy was approximately $30,000 in 2001 and rose to $92,000 in 2012 [2,3]. In addition, various side effects were found in CML patients receiving IM treatment, and dose reduction may help to overcome side effects [4]. Therefore, investigating a new strategy for improving CML therapy is essential.

In CML cells, the BCR-ABL oncoprotein exhibits distinct functions in the cytoplasm and the nucleus. Cytoplasmic BCR-ABL protein is associated with the development of CML via activation of multiple proliferative and anti-apoptotic signaling pathways causing deregulated cell growth [5]. The BCR-ABL protein localizes exclusively in the cell cytoplasm because its kinase domain masks the nuclear localization sequence (NLS); therefore, IM can release the NLS domain to induce BCR-ABL nuclear import [6–8]. Nuclear BCR-ABL can be re-activated either by the removal of IM or through the metabolic decay of IM, and subsequently phosphorylated the Tyr-99 of P73 to trigger apoptosis [9–11]. Altogether, these results suggest that impairment of BCR-ABL nuclear export can induce P73-dependent apoptosis which would be used as a strategy for improving IM efficacy.

The nuclear pore complex (NPC) is a channel across the nuclear membrane that mediates bidirectional transportation [12]. The nuclear protein forms a complex with RanGTP to pass through the NPC, then RanGTP is hydrolyzed to RanGDP by RanGTPase activating protein 1 (RanGAP1), releasing the nuclear protein into the cytoplasm [13]. According to BCR-ABL function, apoptosis induction and anti-apoptosis occur in the nucleus and cytoplasm, respectively [5,14]. Thus, inhibition of BCR-ABL transportation from the nucleus to the cytoplasm might affect the CML cell fate by downregulating RanGAP1 expression.

MicroRNA (miR) is a small noncoding RNA containing approximately 20 nucleotides that can post-transcriptionally regulate the expression of target genes by directly binding to their 3′ untranslated regions (3′ UTRs) [15]. Abnormal expression of miRNAs was observed in numerous tumor malignancies including breast cancer, lung cancer, colon cancer, and leukemia [16]. However, the regulation of RanGAP1 expression by any miRNA in CML cells is still unclear. Therefore, we attempted to distinguish a miRNA regulated RanGAP1 expression and IM efficacy in CML cells through blocking of nuclear BCR-ABL protein export to the cytoplasm.

In the present study, we discovered that miR-1301 can target the *RanGAP1* 3′ UTR. Furthermore, the trend of inverse correlation between the expression level of *RanGAP1* and miR-1301 was demonstrated in CML patients, and RanGAP1 protein downregulation or an increased miR-1301 level is beneficial for the sensitivity of IM to CML cells. Moreover, this study provided experimental evidence establishing that the expression of miR-1301 can induce BCR-ABL nuclear entrapment and P73 transactivation to enhance IM efficacy in CML cells by downregulating RanGAP1 expression, and this can be used as a therapeutic strategy for CML.

## Materials and Methods

### Reagents and antibodies

The RPMI1640 medium and DMEM were purchased from Hyclone (Logan, UT, USA). Penicillin/streptomycin and sodium pyruvate were purchased from Invitrogen (Carlsbad, CA, USA). MTT (3-(4,5-dimethyl-2-thiazolyl)-2,5-dimethyl-2H-tetrazolium bromide) was purchased from Merck (Darmstadt, Germany). IM, Triton X-100, and dimethyl sulfoxide (DMSO) were purchased from Sigma (St. Louis, MO, USA). A protease inhibitor cocktail was purchased from Roche (Boehringer Mannheim, Germany). Polyvinylidene difluoride (PVDF) membranes were purchased from Millipore (Bedford, MA, USA). A protein assay dye reagent was purchased from Bio-Rad Laboratories (Hercules, CA, USA). The mouse anti-RanGAP1, rabbit anti-c-ABL, mouse anti-Bax, rabbit anti-Lamin B, and rabbit anti-phospho-P73 (Tyr-99) antibodies were purchased from Santa Cruz Biotechnology (Santa Cruz, CA, USA); the rabbit anti-glyceraldehyde-3-phosphate dehydrogenase (GAPDH), rabbit anti-poly (ADP-ribose) polymerase (PARP), and rabbit anti-phospho-Crk-like protein (CRKL) (Tyr-207) antibodies were purchased from Cell Signaling Technology (Beverly, MA, USA). Secondary antibodies, including horseradish peroxidase (HRP)-conjugated goat anti-mouse, anti-rabbit immunoglobulin G (IgG), FITC-conjugated goat anti-rabbit IgG, and Fluor 647-conjugated goat anti-mouse IgG, were purchased from Jackson ImmunoResearch (West Grove, PA, USA). Rhodamine phalloidin was purchased from Thermo Scientific (Hemel Hempstead, HS, UK). The Lipofectamine 3000 transfection kit, Opti-MEM, and DAPI stain solution were purchased from Life Technologies (Grand Island, NY, USA).

### Cell culture

The human CML cell lines K562 and KU812 and the human embryonic kidney cell line HEK293 were obtained from the Food Industry Research and Development Institute (Hsinchu, Taiwan). K562 and KU812 cell lines were grown in RPMI1640 supplemented with 10% fetal bovine serum (FBS; Biological Industries, KBH, Israel), 100 units/mL penicillin, and 100 μg/mL streptomycin. The HEK293 cell line was grown in DMEM supplemented with 10% FBS. These cell lines were cultured in a 37°C humidified environment containing 5% CO_2_ and passaged at a ratio of 1:4 every 2 days.

### Patient samples

Blood samples were obtained from four CML patients followed by the Division of Hematology Oncology in the Department of Medicine of Wan Fang Hospital. This study, MicroRNA-1301-mediated RanGAP1 Downregulation Induces BCR-ABL Nuclear Entrapment to Enhance Imatinib Efficacy in Chronic Myeloid Leukemia Cells, was approved by Taipei Medical University-Joint Institutional Review Board (TMU-JIRB). All patients and healthy volunteers in this study signed an informed consent notice that was approved by TMU-JIRB (Approval number 201401003). The peripheral blood mononuclear cells (PBMCs) from four CML patients were used to determine the correlation between the miR-1301 and RanGAP1 mRNA levels in CML cells prepared by gradient centrifugation on a Ficoll-Paque gradient (GE Healthcare Bio-Sciences Corp, NJ, USA) [17]. The PBMCs were lysed in TRIzol reagent (Invitrogen, CA, USA) and stored at −80°C before RNA extraction.

### MTT assay

The MTT assay was used to assess cell viability. Cells were seeded in 6-well plates at a density of 1.5 × 10^5^ cells per well, followed by subsequent treatment with various concentrations of IM for 72 h. Before the end of this treatment, 0.5 mg/mL MTT was added to each well for 1 h. The supernatant was carefully aspirated and formazan crystals were dissolved using DMSO. The absorbance was measured at 570 nm by using a microplate reader (Thermo, MA, USA).

### Annexin V/propidium iodide (PI) staining assay

Apoptosis was analyzed using flow cytometry with annexin V/PI double staining to detect membrane events. In brief, after treatment with IM, whole cells were collected in HEPES buffer (10 mM HEPES at pH 7.4, 140 mM NaCl, and 2.5 mM CaCl_2_). Subsequently, cells were stained with annexin V (2.5 μg/ml) and PI (2 ng/ml) for 20 min, followed by analysis on a flow cytometer (Becton Dickinson, CA, USA) using CellQuest software (Becton Dickinson, CA, USA). The sum of early apoptosis (annexin V^+^/PI^-^) and late apoptosis (annexin V^+^/PI^+^) is presented as total apoptosis.

### RNA extraction and cDNA preparation

CML cells were homogenized in 1 mL of TRIzol reagent according to the manufacturer instructions. Total RNA, including mRNA and miRNA, was purified using Qiagen RNAeasy Columns (Qiagen, Hamburg, Germany). For measuring miRNA levels, cDNA of each miRNA was synthesized with a unique primer (Applied Biosystems) by using 20 ng of the total RNA. For an mRNA quantitative assay, cDNAs were synthesized from 1 μg of total RNA with random primers by using reverse transcriptase (Applied Biosystems). The synthesized cDNA was stored at −80°C for further analysis.

### qRT-PCR

Levels of mature miRNAs and *RanGAP1*, as well as the internal controls *RNU6b* and *GAPDH*, were respectively assayed in triplicate through real-time qRT-PCR assays (Life Technologies, CA, USA). The miRNA and RNU6b levels were measured using Taqman miRNA assays (Life Technologies, CA, USA) according to the manufacturer instructions. The relative expression levels of miRNA and *RanGAP1* were normalized respectively to *RNU6b* and *GAPDH* by using the equation ΔCt = (Ct^miR-1301^ –Ct^U6b^) or (Ct^RanGAP1^ –Ct^GAPDH^). The mean and standard deviation of ΔCt were calculated. Primers used for PCR reaction were *GAPDH* FWD: 5′-GAAGGTGAAGGTCGGAGTC-3′ and *GAPDH* REV: 5′-GAAGATGGTGATGGGATTTC-3′; and *RanGAP1* FWD: 5′-CAGGCTTTCGCTGTCAACC-3′ and *RanGAP1* REV: 5′-GCAGCATCCCTCTTGATTTCAC-3′.

### Immunofluorescence staining

K562 cells were centrifuged on glass slides by using the Shandon CytoSpin 4 (Thermo Fisher Scientific, MA, USA). Cells were fixed on slides with 4% paraformaldehyde and subsequently treated with 0.5% Triton X-100 and preblocked with skimmed milk. Cells were incubated with the indicated antibody at a final dilution of 1:100 in phosphate-buffered saline (PBS). After being washed with PBS, the cells were incubated with a fluorescence dye-conjugated secondary antibody at a 1:100 dilution in PBS and subsequently washed twice with PBS for observation through either confocal microscopy (Leica TCS SP5, Germany) or deconvolution microscopy (Applied Precision DeltaVision, USA). DAPI and rhodamine phalloidin staining were used to determine nuclear DNA and F-actin protein levels, respectively. We quantified the fluorescence intensity of the FITC-conjugated antibody (green, anti-c-ABL) in K562 cells by using the Metamorph Software (Universal Imaging, Downingtown, PA, USA) and normalized it to the nuclear area identified using DAPI staining (blue) to determine the nuclear BCR-ABL protein level [18].

### Subcellular fraction and immunoblotting

Cells were lysed with the modified radioimmunoprecipitation assay buffer (RIPA buffer; 50 mM Tris-HCl at pH 7.4, 5 mM EDTA at pH 7.4, 0.5% Triton X-100, 0.1% sodium deoxycholate, 0.5% NP-40, and 150 mM NaCl) containing a protease inhibitor cocktail for obtaining total cell extracts [19]. To prepare subcellular fraction, cells were resuspended in hypotonic buffer (10 mM HEPES at pH 7.9, 10 mM KCl, 1.5mM MgCl_2_, 0.05% NP40 and 0.5 mM DTT) with a protease inhibitor cocktail on ice for 30 min, then centrifuged to collect supernatant (cytoplasmic fraction). The pellet was washed twice in hypotonic buffer and homogenized by modified RIPA buffer with a protease inhibitor cocktail, then centrifuged to collect supernatant (nuclear fraction). Protein concentrations of total cellular extracts or subcellular fractions were determined using a protein assay kit (Bio-Rad Laboratories, CA, USA). Cell extracts with a sample buffer (0.0625 M Tris-base at pH 6.8, 2% SDS, 10% glycerol, 0.1 M beta-mercaptoethanol, 0.01% bromophenol blue) were placed in boiling water for 5 min and then separated using 10% sodium dodecylsulfate polyacrylamide gel electrophoresis (SDS-PAGE). After electrophoresis, the proteins were transferred to a PVDF membrane for immunoblotting. Membranes were incubated in a 5% skimmed milk solution (blocking solution) for 1 h and then incubated with the indicated antibodies at 4°C for 16 h. The membranes were probed using the appropriate HRP-conjugated secondary antibodies for 1 h at room temperature. Finally, the membrane was visualized through chemiluminescence (Santa Cruz Biotechnology, CA, USA).

### Plasmid transfection

The K562 cell line was transfected with plasmids through electroporation by using the Nucleofector Kit V (Amaxa, Koeln, Germany). K562 cells (10^5^) were resuspended in 100 μL of the nucleofector V solution with 1 μg of RanGAP1 shRNA plasmid, 2.5 μg of miR-455-3p plasmid, or 2.5 μg of miR-1301 plasmid. A luciferase shRNA was used as a scramble control. This nucleofection sample was subsequently transferred to a cuvette and nucleofected with an Amaxa nucleofector apparatus by using the program T-016 and immediately transferred to a 37°C prewarmed culture medium for further testing. Liposome transfection was used for adhesion cells. HEK293 cells were seeded to achieve 70% confluence in 12-well plates without antibiotics. To transfect HEK293 cells with indicated plasmids by Lipofectamine 3000 transfection kit, 0.75 μl Lipofectamine 3000 reagent was mixed with 25 μl Opti-MEM, and 0.25 μg of miR-1301 plasmid or 0.5 μg of *RanGAP1*-3′ UTR-containing plasmid were diluted in 25 μl Opti-MEM with 2 μl P3000 reagent; these two solutions were subsequently mixed and incubated at room temperature for 15 min. The mixture was added to 5 x 10^4^ HEK293 cells and incubated for 6 h, and the medium was refreshed for further testing.

### Luciferase reporter assay

HEK293 cells were plated in 12-well microplates at a concentration of 5 × 10^4^ cells per well the day before transfection. After transfection for 24 h, the cells were washed using PBS and lysed in Passive Lysis Buffer (Promega, WI, USA). The luciferase activity was monitored using a luciferase assay kit (Promega, WI, USA). Light emission was determined using a luminometer (Turner Designs, CA, USA), and each experiment was performed in triplicate.

### Microarray analysis and miRNA prediction

The complete microarray (mRNA and miRNA) dataset is available through the Gene Expression Omnibus (GEO) of the NCBI (http://www.ncbi.nlm.nih.gov/geo/). The potential miRNA binding sites in the *RanGAP1* 3′ UTR were searched using TargetScan to predict miRNA targets [20].

### Statistical analysis

Values are presented as the mean + standard deviation (SD). Data comparisons between two groups were performed using Student’s *t* test. A one-way analysis of variance (ANOVA) followed by Tukey’s post hoc test was performed when three or more groups were analyzed. A value of *p* < 0.05 was considered statistically significant.

## Results

### Increased RanGAP1 levels in CML cells

**Fig 1:**
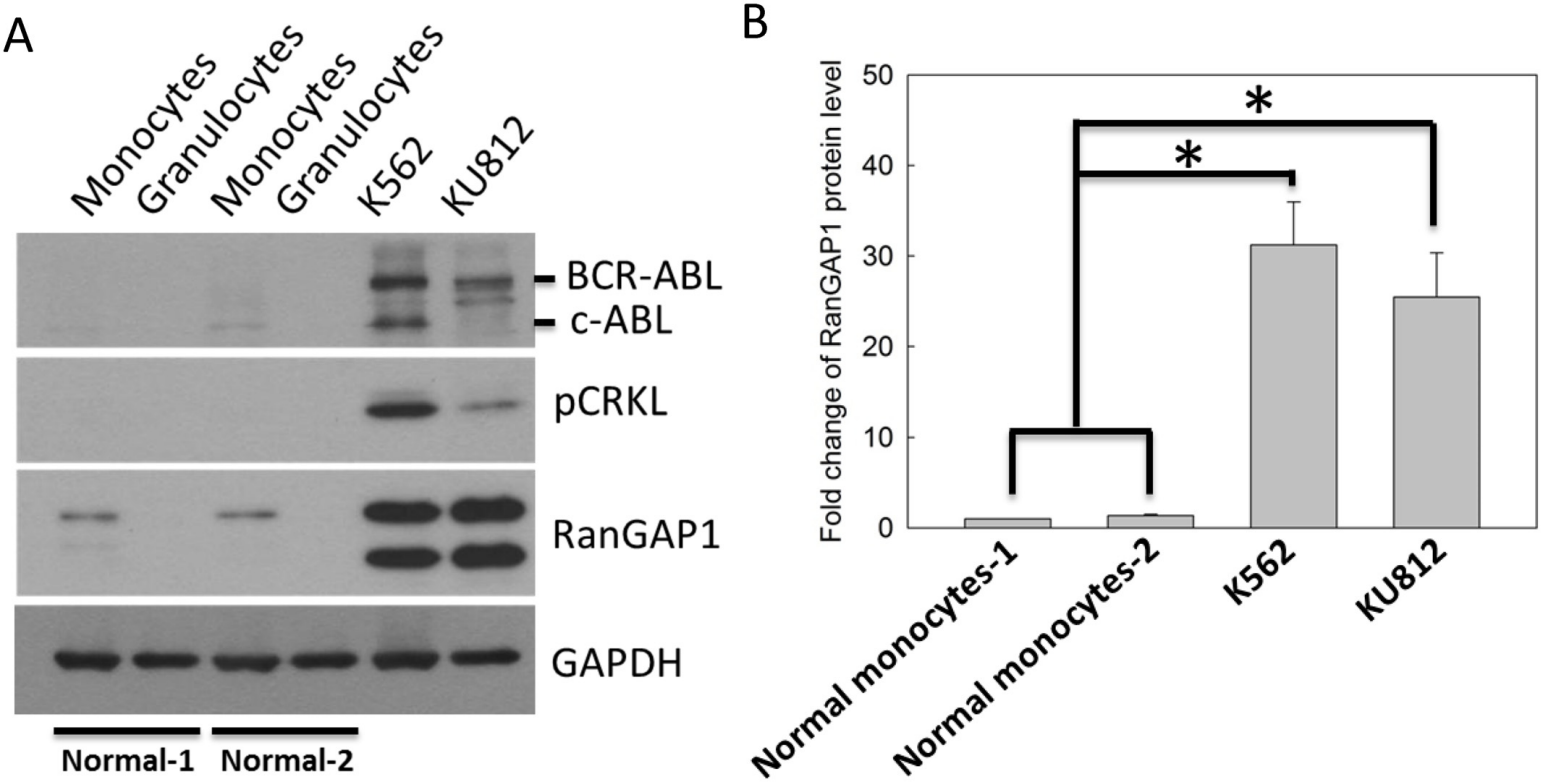
RanGAP1 protein level was increased in CML cells compared with that in normal cells. (A) The RanGAP1 protein levels were measured using an immunoblot assay in normal granulocytes and monocytes from healthy volunteers, and CML cell lines. GAPDH was used as an internal control. Phosphorylated CRKL (Tyr-207) is a marker of active BCR-ABL in CML cells. (B) The quantitative data indicate the RanGAP1 protein levels in normal monocytes and CML cell lines from (A). Results are presented as means + SD of three independent experiments. *p < 0.05, compared with the control.

In Fig 1A, we analyzed the RanGAP1 protein level in normal granulocytes and monocytes from healthy volunteers (S1 Table), and CML cell lines. The BCR-ABL oncoprotein and its substrate, phosphorylated CRKL (Tyr-207), were markers of CML cells (Fig 1A). The immunoblot data revealed that the BCR-ABL, phosphorylated CRKL, and RanGAP1 protein levels were significantly increased in K562 and KU812 cell lines compared with those in normal monocytes or granulocytes (Fig 1A and 1B). These data demonstrated that the protein levels of RanGAP1 was increased in CML cells, suggesting that RanGAP1 may be crucial in CML.

### Downregulation of the RanGAP1 level enhances IM efficacy in K562 cells

**Fig 2:**
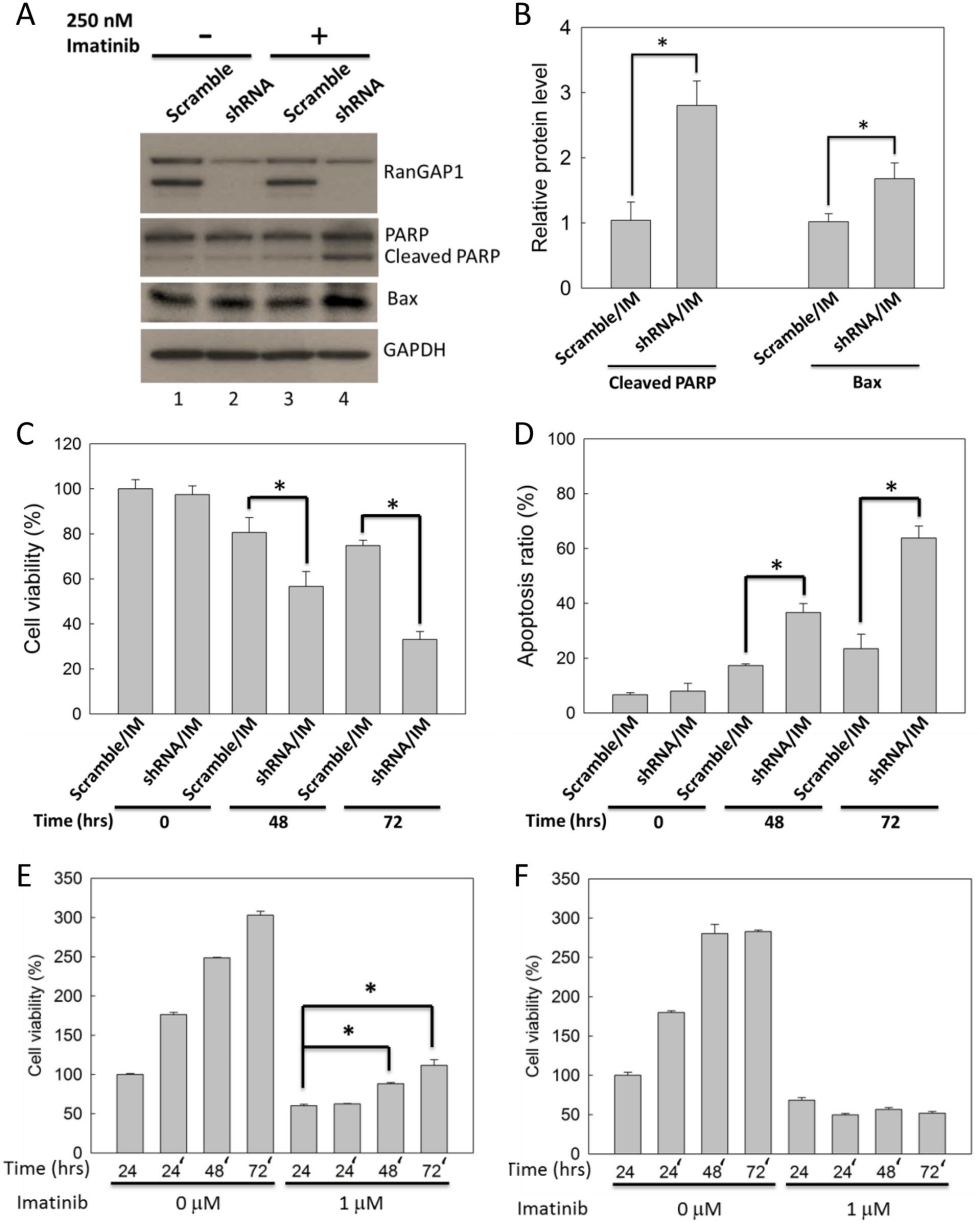
RanGAP1 downregulation enhanced and prolonged IM-induced cytotoxicity effect in K562 cells. K562 cells were transfected with RanGAP1 shRNA and treated with 250 nM IM. (A) The immunoblot assay was conducted at 48 h. GAPDH was used as an internal control. (B) The bar graph indicates quantitative data of relative cleaved PARP/GAPDH and Bax/GAPDH levels from (A). The cell viability (C) and apoptosis ratio (D) of K562 cells were measured by MTT assay and by annexin V/PI staining, respectively, at 48 or 72 h. PI, means propidium iodide. The results are presented as means + SD of three independent experiments. *p < 0.05, compared with the scramble group receiving IM treatment. Scramble plasmid- (E) and RanGAP1 shRNA- (F) transfected K562 cells were treated with 1 μM IM for 24 h and then washed with phosphate buffer saline and plated in a fresh culture medium for extend 72 h; subsequently, an MTT assay was performed to assess cell viability. The results are presented as means + SD of three independent experiments. *p < 0.05, compared with the scramble group receiving IM treatment at 24 h.

K562 cells were transfected with RanGAP1 shRNA and subsequently treated with 250 nM IM for 48 h to examine whether downregulation of the RanGAP1 protein level enhances drug efficacy in CML cells. RanGAP1 protein level was not affected by IM in K562 cells (Fig 2A, lane 1 and 3). IM-treated K562 cells with RanGAP1 knockdown exhibited increased protein levels of the cleaved form of PARP and Bax compared with those in the scramble group (Fig 2A, lane 3 and 4, and 2B). In addition, RanGAP1 knockdown enhanced IM-reduced K562 cells viability by approximately 20% and 40% for 48 h and 72 h (Fig 2C), and also enhanced IM-induced K562 cells apoptosis by approximately 20% and 40% for 48 h and 72 h (Fig 2D). Furthermore, after 1 μM IM pretreatment for 24 h followed by drug withdrawal for another 72 h, K562 cell viability in the RanGAP1 knockdown group was not reversed as it was in the scramble group (Fig 2E and 2F). Altogether, these data demonstrate that downregulation of the RanGAP1 protein level enhanced IM efficacy and prolonged the IM-induced cell viability reduction effect in K562 cells.

### RanGAP1 affects BCR-ABL protein distribution in IM-treated K562 cells

**Fig 3:**
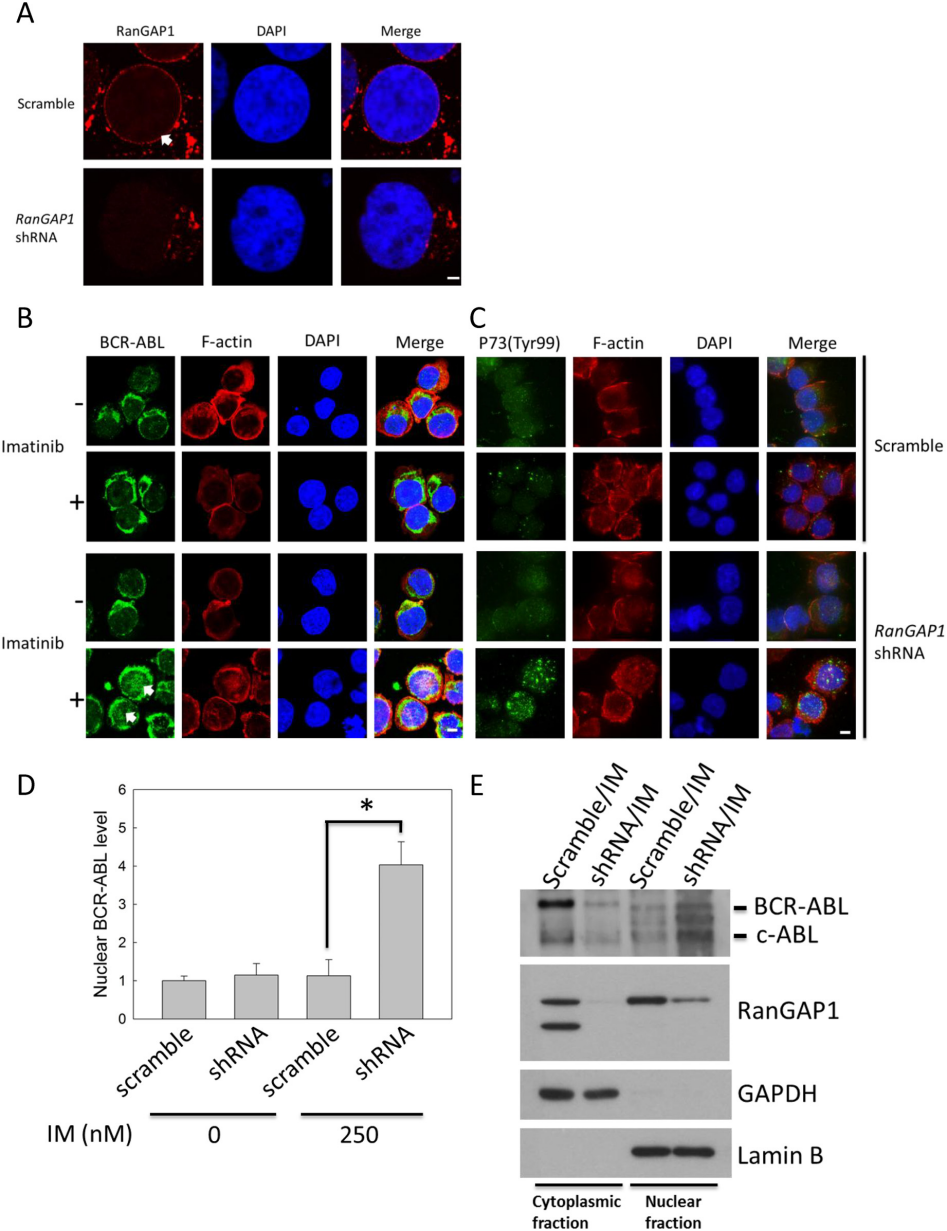
Combination of RanGAP1 knockdown and IM treatment induced nuclear entrapment of BCR-ABL and increased the phosphorylation level of P73 in K562 cells. K562 cells were transfected with a scramble plasmid or RanGAP1 shRNA and subsequently treated with 250 nM IM for 48 h. The RanGAP1 (A, red) and BCR-ABL protein levels (B, green) and P73 phosphorylation state (Tyr-99) (C, green) were observed by using immunofluorescence staining through confocal microscopy as described in materials and methods. (D) The quantitative data indicate the nuclear BCR-ABL level in K562 cells (cell number = 15) from (B). The results are presented as means + SD. *p < 0.05, compared with the scramble group receiving IM treatment at 48 h. Arrows denote the position of indicated proteins in K562 cells. Scale bars = 25 μm (A). Scale bars = 75 μm (B, C). (E) The K562 cell lysates were separated by cytoplasmic and nuclear fractions, and then were analyzed by immunoblot with anti-c-ABL, anti-RanGAP1, anti-GAPDH, and anti-Lamin B antibodies. GAPDH and Lamin B were used as internal controls for cytoplasmic and nuclear fraction, respectively.

The BCR-ABL oncoprotein, which proceeds to the nucleus from the cytoplasm on IM treatment, induces CML cell apoptosis [8]. We used immunofluorescence staining to detect RanGAP1 and BCR-ABL protein localization in K562 cells for identifying whether RanGAP1 downregulation enhanced IM efficacy in these cells by influencing BCR-ABL subcellular localization. The data revealed that RanGAP1 was concentrated at the nuclear membranes in the scramble group and was decreased in the RanGAP1 shRNA transfection group (Fig 3A). RanGAP1 knockdown had no effect on BCR-ABL distribution in K562 cells (Fig 3D). However, in RanGAP1-knockdown K562 cells, IM treatment induced a great amount of BCR-ABL to be accumulated in the nucleus (Fig 3B) and increased the P73 phosphorylation level on Tyr-99 residue (Fig 3C). The nuclear BCR-ABL protein level was increased by approximately three-fold compared with that in the vector control group (Fig 3D). Moreover, we also used the immunoblot assay to confirm that BCR-ABL protein was detected in the nuclear fraction of K562 cells after RanGAP1 downregulation with IM treatment (Fig 3E). Hence, combination of RanGAP1 downregulation and IM treatment may impair BCR-ABL nucleo-cytoplasmic shuttling, which induces P73 phosphorylation in K562 cells.

### Decreased *RanGAP1*-targeting miRNA in CML cells

**Fig 4:**
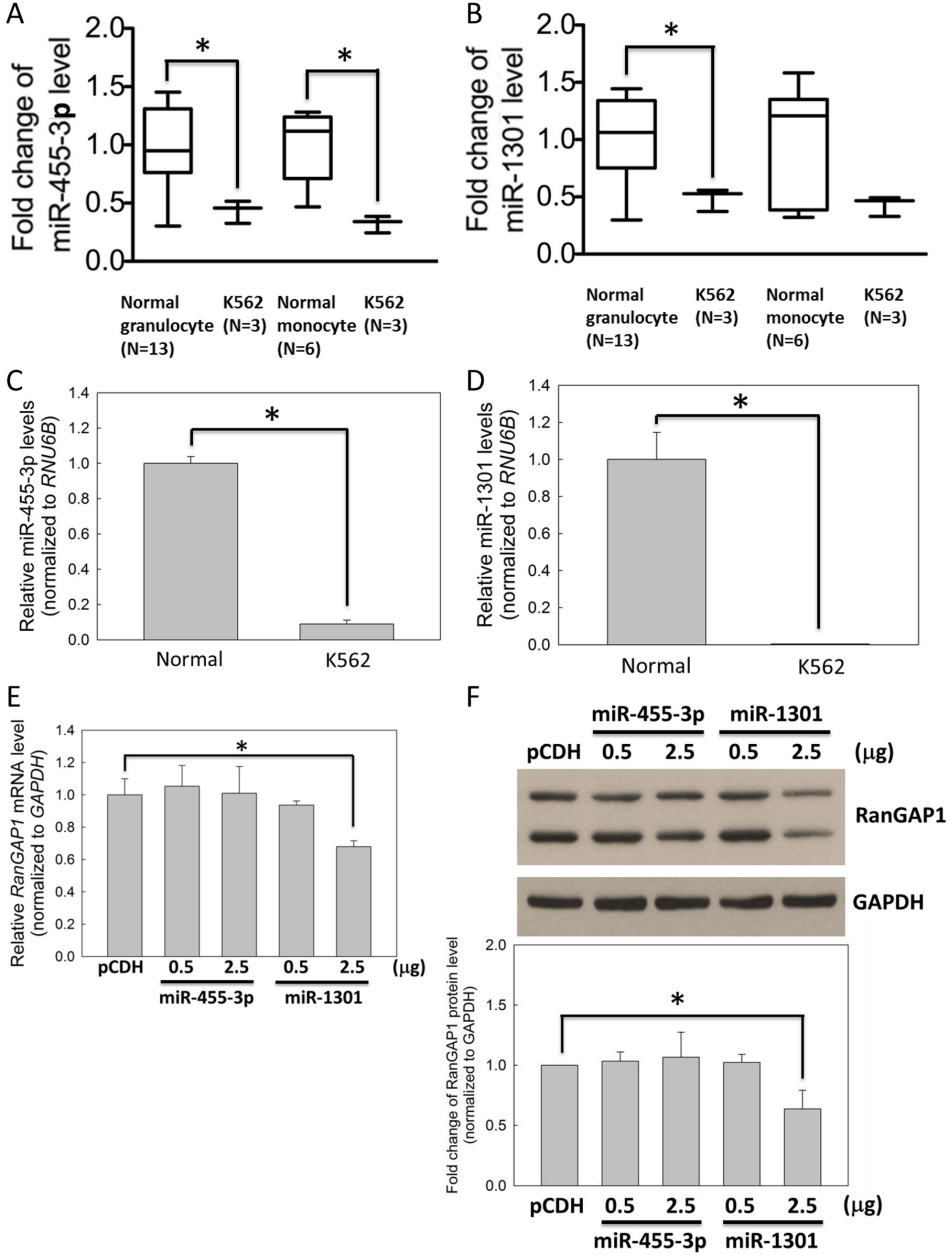
Expression level of miR-1301 was lower in CML cells compared with that in normal cells. The mRNA expression levels of miR-455-3p (A) and miR-1301 (B) in K562 cells compared with those in normal monocytes and granulocytes from healthy volunteers were measured using microarrays from the GEO database (accession number GSE51908). In addition, the expression levels of miR-455-3p (C) and miR-1301 (D) in K562 cells were measured through qRT-PCR relative to those in normal monocytes. K562 cells were transfected with pCDH (vector only), miR-455-3p, or miR-1301 plasmids for 48 h and subsequently subjected to qRT-PCR (E) and immunoblot assays (F) for assessing the RanGAP1 level. (F) The bar graph indicates quantitative data on the RanGAP1 protein level. GAPDH was used as an internal control. Results are presented as means + SD of three independent experiments. *p < 0.05, compared with the control.

Previous study demonstrated that abnormal levels of miRNA were observed in tumor malignancy [16]. This study revealed that RanGAP1 protein level affects IM efficacy in K562 cells (Fig 2C and 2D). However, whether any miRNA regulates *RanGAP1* expression is still unclear. According to the microarray data from GEO database (accession number GSE51908) [21], twenty-four miRNAs were downregulated in K562 cells compared with normal monocytes and granulocytes from healthy volunteers (fold change ≦ 0.5; *p* value ≦ 0.05) (S1A Fig). Of which, only miR-455-3p and miR-1301 were predicted to target to the *RanGAP1* 3′ UTR by TargetScan (S1B Fig), and both were significantly downregulated in K562 cells compared with normal monocytes and granulocytes (Fig 4A and 4B). In addition, compared with normal monocytes, the qRT-PCR data revealed that the miR-455-3p and miR-1301 levels were reduced in K562 cells by approximately 10- and 100-fold (Fig 4C and 4D). Moreover, the mRNA and protein levels of RanGAP1 were downregulated significantly by miR-1301 compared with miR-455-3p in K562 cells examined through qRT-PCR (Fig 4E) and immunoblotting (Fig 4F), respectively. Therefore, we further focused on the effects of miR-1301 in the subsequent studies.

### Correlation between miR-1301 and *RanGAP1* levels in CML cells

**Fig 5:**
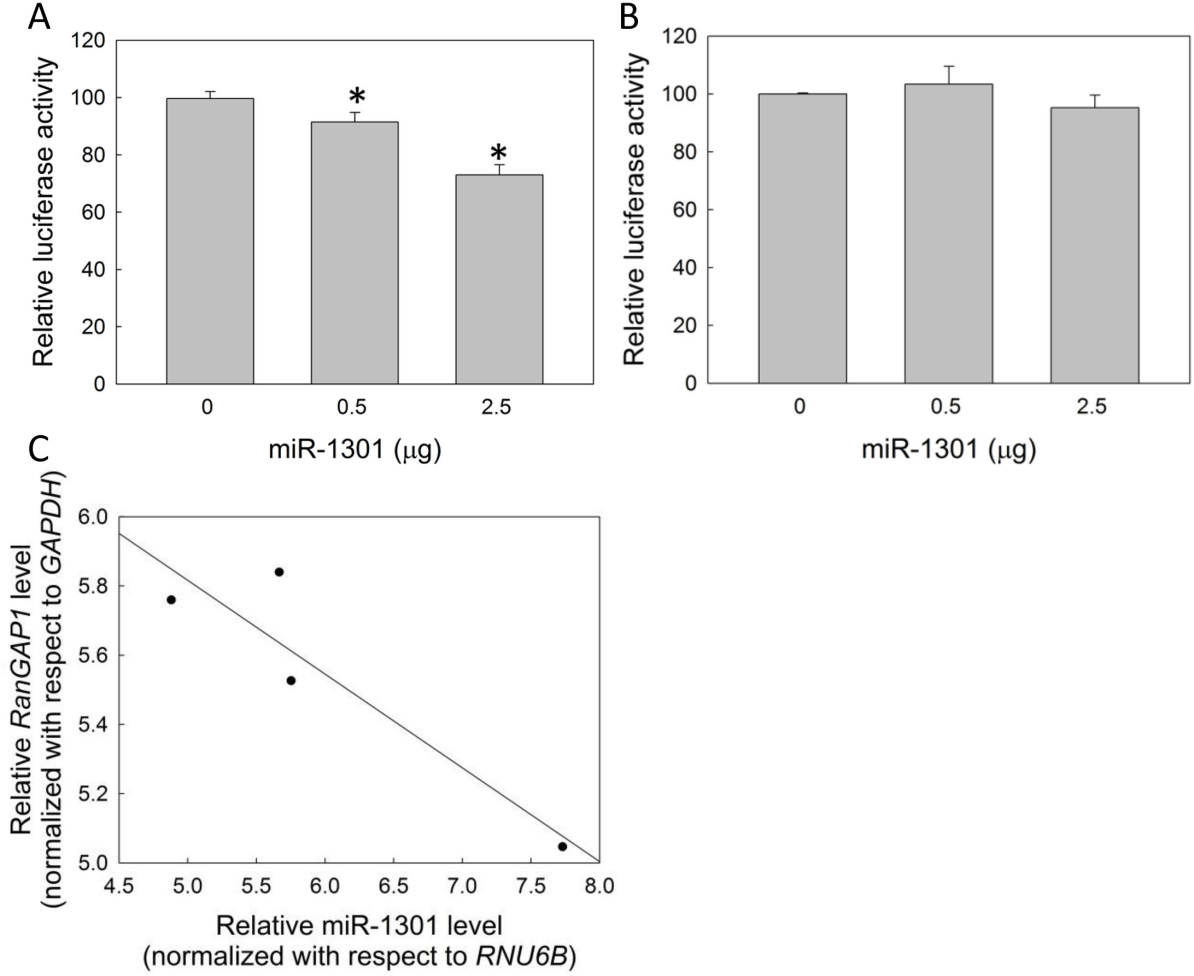
MiR-1301 targets *RanGAP1* via the 3′ UTR. Reporter constructs containing the wild type (A) or mutant type (B) *RanGAP1* 3′ UTR were cotransfected with the miR-1301 plasmid in HEK293 cells. The relative luciferase activity was normalized to Renilla luciferase. The results are presented as means + SD of three independent experiments. *p < 0.05, compared with the control. (C) The expression levels of miR-1301 and *RanGAP1* are shown relative to those of *RNU6B* and *GAPDH*, respectively, in 4 CML patients. The coefficient of determination (R^2^), Pearson product-moment correlation coefficient (R), and *p* value of the regression line were 0.826, 0.922, and 0.078, respectively.

We generated luciferase reporter constructs containing the *RanGAP1* 3′ UTR with either wild or mutant type binding sites for miR-1301 in HEK293 cells (S1B Fig) to confirm that miR-1301 reduces *RanGAP1* expression via the 3′ UTR. Reducing luciferase activity implied that miR-1301 could interact with the *RanGAP1* 3′ UTR (Fig 5A), whereas mutant type miR-1301 binding sites in the *RanGAP1* 3′ UTR abolished miR-1301 binding ability (Fig 5B). In addition, a trend of inverse correlation between *RanGAP1* and miR-1301 levels was observed in CML patients (coefficient of determination, R^2^ = 0.826; Pearson product-moment correlation coefficient, R = 0.922; *p* = 0.078) (Fig 5C and S2 Table). These data suggested that miR-1301 can downregulate *RanGAP1* expression in CML cells.

### MiR-1301 enhances IM efficacy in K562 cells

**Fig 6:**
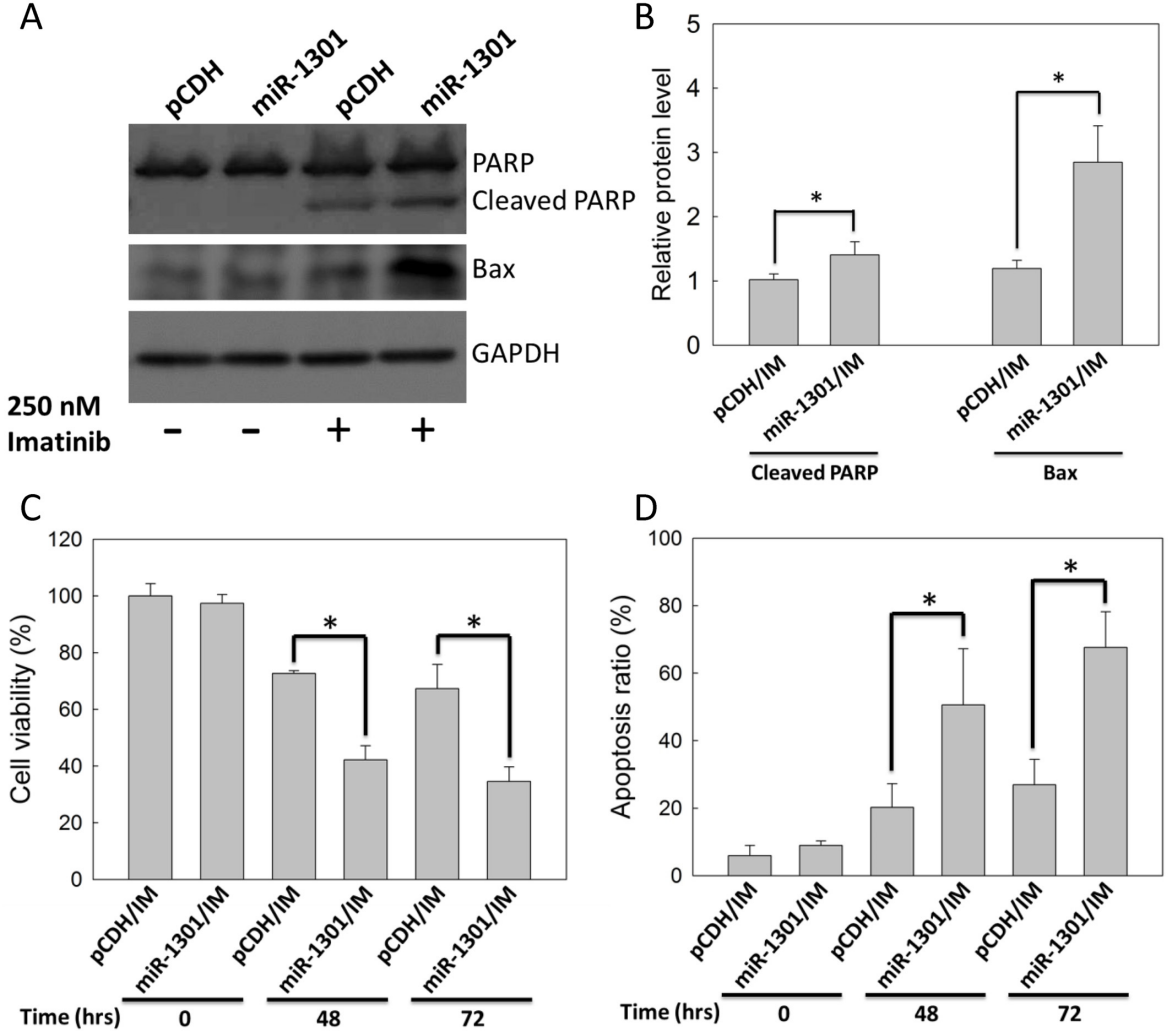
MiR-1301 enhanced IM efficacy in K562 cells. K562 cells were treated with 250 nM IM after transfection with pCDH (vector only) or the miR-1301 plasmid. (A) The immunoblot assay was conducted at 48 h. GAPDH was used as an internal control. (B) The bar graph indicates quantitative data of relative cleaved PARP/GAPDH and Bax/GAPDH levels from (A). The cell viability (C) and apoptosis ratio (D) of K562 cells were measured by MTT assay and by annexin V/PI staining, respectively, at 48 or 72 h. Results are presented as means + SD of three independent experiments. *p < 0.05, compared with the vector group (pCDH) receiving IM treatment.

K562 cells were transfected with the miR-1301 plasmid and subsequently treated with 250 nM IM for 48 h to examine whether miR-1301 enhances IM efficacy in CML cells. IM-treated K562 cells with miR-1301 plasmid transfection exhibited higher protein levels of the cleaved form of PARP and Bax compared with those in the vector group (Fig 6A and 6B). In addition, miR-1301 enhanced IM-reduced K562 cells viability by approximately 30% and 40% at 48 h and 72 h (Fig 6C), and also enhanced IM-induced K562 cells apoptosis by approximately 30% and 45% at 48 h and 72 h (Fig 6D). Altogether, these data demonstrated that miR-1301 could enhance IM efficacy in K562 cells.

### MiR-1301 affects BCR-ABL distribution in IM-treated K562 cells by downregulating RanGAP1 expression

**Fig 7:**
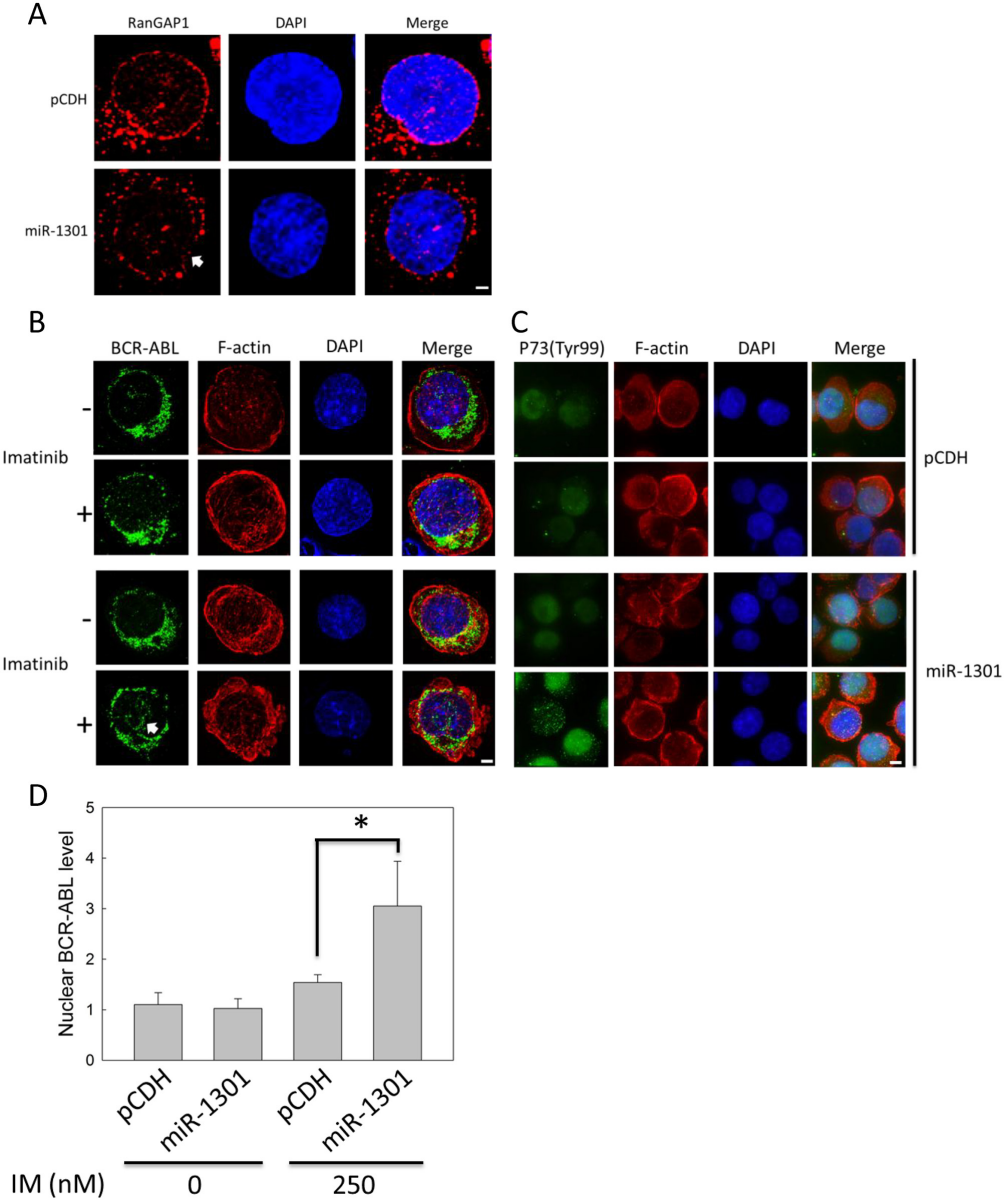
Combination of RanGAP1 knockdown by miR-1301 and IM treatment induced nuclear entrapment of BCR-ABL and increased P73 phosphorylation levels in K562 cells. K562 cells were transfected with pCDH (vector only) or the miR-1301 plasmid and subsequently treated with 250 nM IM for 48 h. The RanGAP1 (A, red) and BCR-ABL protein levels (B, green) and P73 phosphorylation state (Tyr-99) (C, green) were observed using immunofluorescence staining through deconvolution microscopy as described in materials and methods. (D) The quantitative data indicate the nuclear BCR-ABL level in K562 cells (cell number = 15) from (B). The results are presented as means + SD. *p < 0.05, compared with the pCDH (vector only) receiving IM treatment. Arrows denote the position of indicated proteins in K562 cells. Scale bars = 25 μm (A, B). Scale bars = 75 μm (C).

We used immunofluorescence staining to detect RanGAP1 and BCR-ABL protein in K562 cells for determining whether miR-1301 downregulates RanGAP1 to enhance IM efficacy by inducing BCR-ABL nuclear entrapment. The data revealed that miR-1301 reduced the RanGAP1 level compared with that in the vector group (Fig 7A). RanGAP1 knockdown by miR-1301 had no effect on BCR-ABL distribution in K562 cells (Fig 7D). However, transfecting IM-treated K562 cells with the miR-1301 plasmid induced a great amount of BCR-ABL to accumulate in the nucleus (Fig 7B and S1 File). The nuclear BCR-ABL protein level was increased by approximately two-fold compared with that in the vector control group (Fig 7D). Moreover, miR-1301 increased the P73 phosphorylation level on Tyr-99 residue in IM-treated K562 cells (Fig 7C). Therefore, combination of miR-1301-induced RanGAP1 downregulation and IM treatment may block BCR-ABL nucleo-cytoplasmic shuttling to enhance the P73 phosphorylation level in K562 cells.

## Discussion

In the present study, we demonstrated that downregulation of RanGAP1 impaired BCR-ABL nuclear export and enhanced IM efficacy in K562 cells. Our data revealed that RanGAP1 knockdown may enhance IM efficacy by inducing BCR-ABL accumulation in the nucleus and that RanGAP1 plays a regulatory role in BCR-ABL transportation and IM efficacy to determine the fate of CML cells. MiR-1301 is a *RanGAP1*-targeting miRNA, and the trend of inverse correlation between miR-1301 and the *RanGAP1* level is observed in CML patients. Therefore, miR-1301-mediated RanGAP1 knockdown may be a useful strategy for enhancing IM efficacy.

The RanGAP1 protein level correlated with IM drug efficacy in CML cells. The protein levels of RanGAP1 was increased in CML cell lines compared with those in normal cells (Fig 1A and 1B). RanGAP1 downregulation enhanced IM drug efficacy and prolonged IM-induced cell viability reduction in K562 cells through RanGAP1 shRNA transfection (Fig 2C–2F). A previous study revealed that the ectopic expression of wild type RanGAP1 elicits IM drug resistance in K562 cells [22]. Moreover, RanGAP1 knockdown induced lymphoma cell death and cell-cycle arrest [23]. These studies imply that RanGAP1 is a crucial target protein for tumor therapy, and downregulation of RanGAP1 is beneficial in enhancing IM efficacy in CML cells.

RanGAP1 downregulation impairs BCR-ABL oncoprotein nuclear export to induce cell apoptosis and can thus be used as a therapeutic strategy for CML. Combination of RanGAP1 knockdown by shRNA or miR-1301 with IM treatment substantially induced BCR-ABL accumulation in the K562 cell nucleus (Figs 3B and 7B) and significantly enhanced IM-induced apoptosis in K562 cells (Figs 2D and 6D). Previous studies have reported results similar to those of our study evidencing that nuclear entrapment of BCR-ABL enhances IM efficacy in CML cells. RanGAP1 hydrolyzes RanGTP to RanGDP and then dissociates RanGDP from adaptor protein chromosomal maintenance 1 (CRM1) and releases nuclear protein into the cytoplasm [13]. Leptomycin B (LMB), a nuclear export inhibitor, has been reported to inhibit adaptor protein CRM1 binding with the nuclear protein-RanGTP complex [7,24–26]. K562 cells co-treated with IM and LMB exhibited BCR-ABL localization in the nucleus predominantly, and were more effective in inducing K562 cell apoptosis compared with single IM treatment; these results were also demonstrated by *ex vivo* models [10,27,28]. However, the therapeutic application of LMB is limited by its neuronal toxicity [29]. These results indicate that nuclear BCR-ABL-induced apoptosis is depended on regulation of the RanGTPase system, and RanGAP1 downregulation may inhibit BCR-ABL nuclear transportation to the cytoplasm by reducing the RanGTP hydrolysis rate. Both miR-1301 and RanGAP1 shRNA significantly enhanced IM efficacy in K562 cells (Figs 2D and 6D), but miR-1301-induced RanGAP1 knockdown was much less than that induced by RanGAP1 shRNA proved by immunoblotting (Figs 2A and 4F) and immunofluorescence staining (Figs 3A and 7A). Therefore, we could not exclude the possibility that the enhancement of IM efficacy by miR-1301 in CML cells may result from other signaling pathways. Moreover, further investigation regarding whether miR-1301 induces side effects in normal cells is necessary.

P73 protein is involved in the nuclear BCR-ABL-induced apoptosis pathway. Combination of RanGAP1 knockdown with IM treatment induced substantial BCR-ABL protein accumulation in the nucleus (Figs 3B and 7B) and increased the phosphorylation level of P73 (Tyr-99) in K562 cells (Figs 3C and 7C). P73 is activated by c-ABL on Tyr-99 phosphorylation, a prerequisite modification for P73 to induce cell death in fibroblasts [30]. These results suggest that RanGAP1 downregulation can induce P73 phosphorylation on Tyr-99 residue via nuclear BCR-ABL. In our study, RanGAP1 knockdown by shRNA or miR-1301 increased the protein levels of the cleaved form of PARP and Bax in IM-treated K562 cells (Figs 2A and 6A). Activation of P73 and its downstream pathway has been reported to induce CML cell apoptosis, which is triggered by nuclear BCR-ABL [11,31]. P73 binds and transactivates the Bax gene promoter to induce apoptosis in irradiated T cells and ovarian cancer cells [32,33]. Altogether, these results imply that miR-1301-mediated RanGAP1 knockdown induces BCR-ABL nuclear entrapment to enhance IM efficacy in K562 cells via activation of P73 and its downstream pathway.

**Fig 8:**
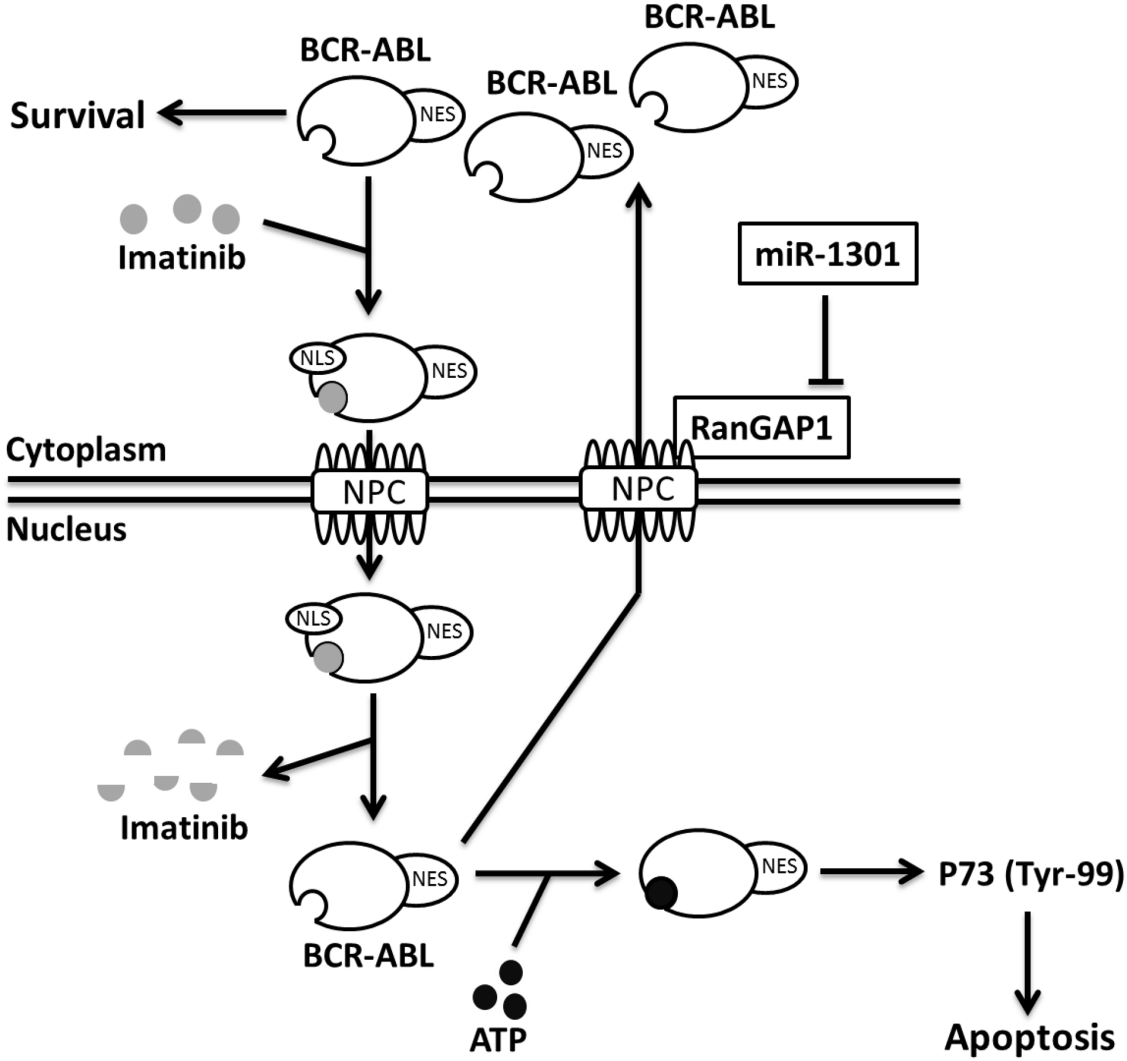
Proposed mechanism of IM efficacy enhancement. MiR-1301-inhibited RanGAP1 expression reduces BCR-ABL nucleo-cytoplasmic shuttling; therefore, nuclear BCR-ABL activates P73 and increases IM-induced apoptosis in CML cells. NES, nuclear export signal. NLS, nuclear localization signal. NPC, nuclear pore complex.

In CML cells, miR-1301 is a tumor suppressor, and its mRNA level is reduced in K562 cells but increased in normal monocytes (Fig 4B and 4D). Aberrant miRNA expression was observed in numerous cancers compared with their normal tissue counterparts [34]. In CML cells, miRNAs were aberrantly over- or under- expressed and were reported to act as tumor suppressors [35]. However, whether miR-1301 level is regulated by IM treatment or other therapy in CML cells needs to be further elucidated. The mean steady state C_min_ of IM in CML patients with 400 mg dose daily is approximately 800–2400 nM [36]. Bernardo et al. demonstrated that the cell viability were reduced by approximately 30% and 70% respectively by 250 and 1000 nM IM in K562 cells at 48 h [37]. In our study, 1000 nM IM reduced approximately 60% K562 cell viability at 48 h (S2 Fig). Moreover, 250 nM IM treatment alone or combination with miR-1301 transfection respectively reduced K562 cells viability by approximately 30% and 60% and also induced K562 cells apoptosis by approximately 20% and 60% at 48 h (Fig 6C and 6D). These results indicate that miR-1301 transfection with lower concentrations of IM inducing the better cytotoxicity to CML cells as higher concentration of IM treatment is clinically relevant. In this study, we demonstrated for the first time that miR-1301 directly downregulates RanGAP1 expression to suppress BCR-ABL nucleo-cytoplasmic shuttling and induces P73 phosphorylation in K562 cells, and it subsequently sensitizes these cells to IM treatment (Fig 8). The trend of inverse correlation between *RanGAP1* and miR-1301 mRNA levels was observed in CML patients (Fig 5C). Furthermore, RanGAP1 protein was also expressed in CML patients (S3 Fig). Therefore, upregulation of miR-1301 expression may have therapeutic efficacy against CML. However, the function and underlying mechanisms of miR-1301 in the initiation and progression of CML still warrant further investigation.

In conclusion, we revealed that miR-1301 induced nuclear entrapment of BCR-ABL and enhanced IM efficacy in CML cells by downregulating the RanGAP1 protein level. Furthermore, we demonstrated that the levels of RanGAP1 protein and miR-1301 are correlated with IM efficacy in CML cells. The ectopic expression of miR-1301 can be used in combination with lower concentrations of IM for treating CML patients, thereby minimizing the side effects and inducing the better cytotoxicity to CML cells as higher concentration of IM treatment. Our study suggests that RanGAP1 downregulation by miR-1301 or other methods is a novel beneficial strategy for enhancing IM efficacy in CML patients.

## Supporting Information

S1 Fig RanGAP1 expression-associated miRNA level was decreased in K562 cells compared with that in normal monocytes and granulocytes. (A) The list of twenty-four miRNAs significantly downregulated in K562 cells respectively compared to normal monocytes and granulocytes from healthy volunteers (FC ≦ 0.5; *p* value ≦ 0.05) according to the microarray data from GEO database (accession number GSE51908). FC, fold change. *p* value, compared with respective normal cells. *p value < 0.05, **p value < 0.01, ***p value < 0.001. (B) Schematic of the *RanGAP1* 3′ UTR binding site for miR-455-3P and miR-1301, and mutant type *RanGAP1* 3′ UTR constructs on the miR-1301 binding site.
(TIFF)

S2 Fig IM decreased K562 cells viability in a dose dependent manner. The viability of K562 cells was measured by an MTT assay after treatment with the indicated doses of IM for 48 h. The results are presented as means + SD of three independent experiments. Data abcdef without the same letter are significantly different from each group (p < 0.05).
(TIFF)

S3 Fig RanGAP1 protein was expressed in CML cells. The RanGAP1 protein levels were measured using an immunoblot assay in granulocytes and monocytes from CML patient, and K562 cells. GAPDH was used as an internal control. The CRKL phosphorylation level on Tyr-207 is activated by BCR-ABL, which is used as a marker of CML cells.
(TIFF)

S1 File Combination of RanGAP1 knockdown by miR-1301 and IM treatment significantly induced BCR-ABL nuclear entrapment in miR-1301-transfected K562 cells. K562 cells were transfected with pCDH (vector only) or the miR-1301 plasmid and subsequently treated with 250 nM IM for 48 h. The protein levels were observed using immunofluorescence staining through deconvolution microscopy as described in materials and methods. Video of various z-stack data from K562 cells expressing BCR-ABL (green) colabeled with the nuclear dye DAPI (blue).
(PPTX)

S1 Table Basic clinical parameters of the healthy volunteers involved in the study. (TIFF)

S2 Table Basic clinical parameters of the CML patients involved in the study. (TIFF)
