# Correction: MicroRNA-1301-Mediated RanGAP1 Downregulation Induces BCR-ABL Nuclear Entrapment to Enhance Imatinib Efficacy in Chronic Myeloid Leukemia Cells

**DOI:** 10.1371/journal.pone.0173828

**Published:** 2017-03-07

**Authors:** Tsung-Yao Lin, Ku-Chung Chen, Hsing-Jin Eugene Liu, Ann-Jeng Liu, Kun-Li Wang, Chwen-Ming Shih

There is an error in the second affiliation for authors Tsung-Yao Lin, Ku-Chung Chen, and Chwen-Ming Shih. The correct affiliation is: Department of Biochemistry and Molecular Cell Biology, School of Medicine, College of Medicine, Taipei Medical University, Taipei, Taiwan.
