# Supplementary figures and images for: MicroRNA-1301-Mediated RanGAP1 Downregulation Induces BCR-ABL Nuclear Entrapment to Enhance Imatinib Efficacy in Chronic Myeloid Leukemia Cells
Source: PLoS One. 2016 May 26;11(5):e0156260. doi: 10.1371/journal.pone.0156260 (PMC4881950; doi:10.1371/journal.pone.0156260)

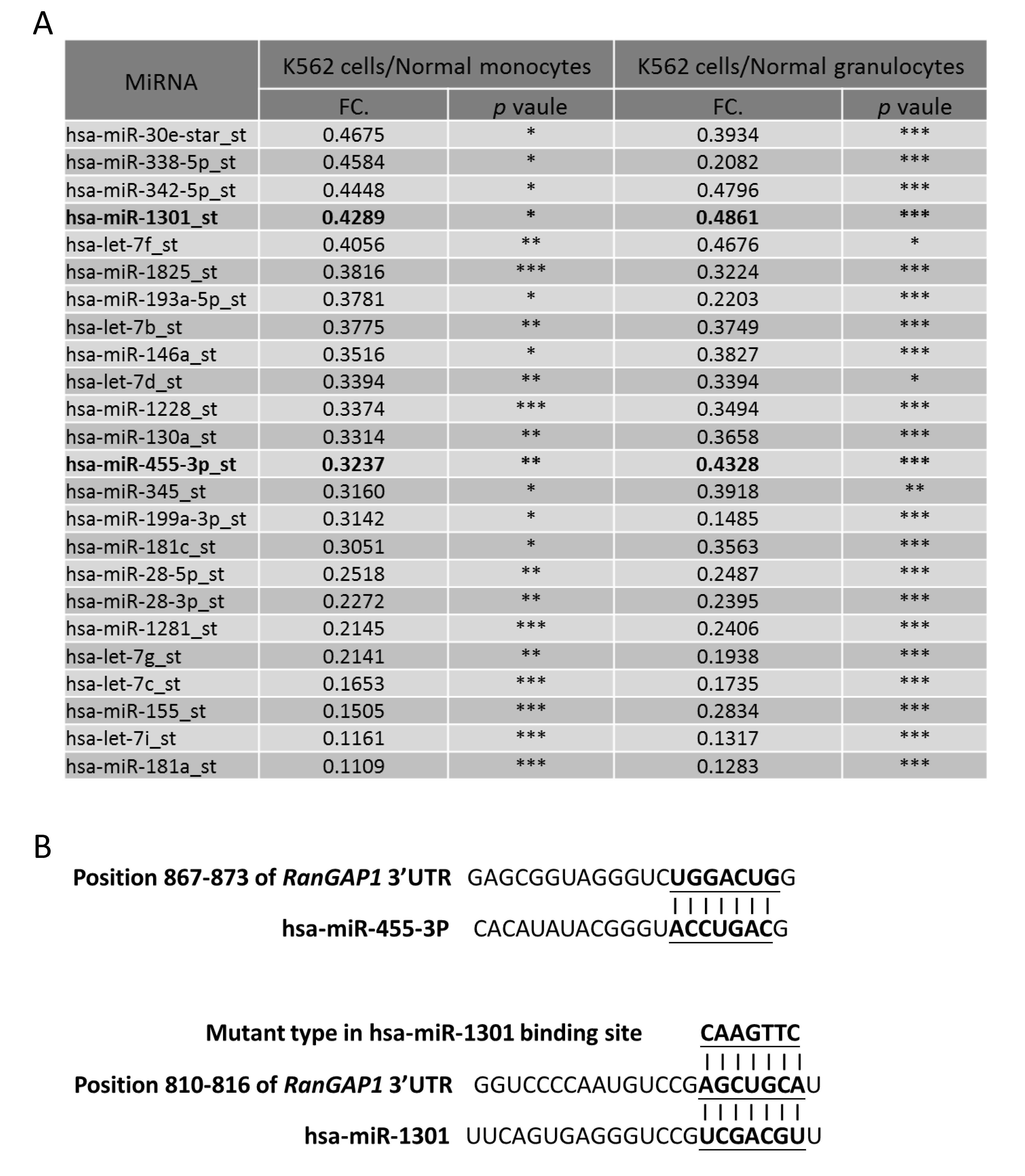

Supplement: S1 Fig — (A) The list of twenty-four miRNAs significantly downregulated in K562 cells respectively compared to normal monocytes and granulocytes from healthy volunteers (FC ≦ 0.5; p value ≦ 0.05) according to the microarray data from GEO database (accession number GSE51908). FC, fold change. p value, compared with respective normal cells. *p value < 0.05, **p value < 0.01, ***p value < 0.001. (B) Schematic of the RanGAP1 3′ UTR binding site for miR-455-3P and miR-1301, and mutant type RanGAP1 3′ UTR constructs on the miR-1301 binding site. (TIFF) [file pone.0156260.s001.tiff]

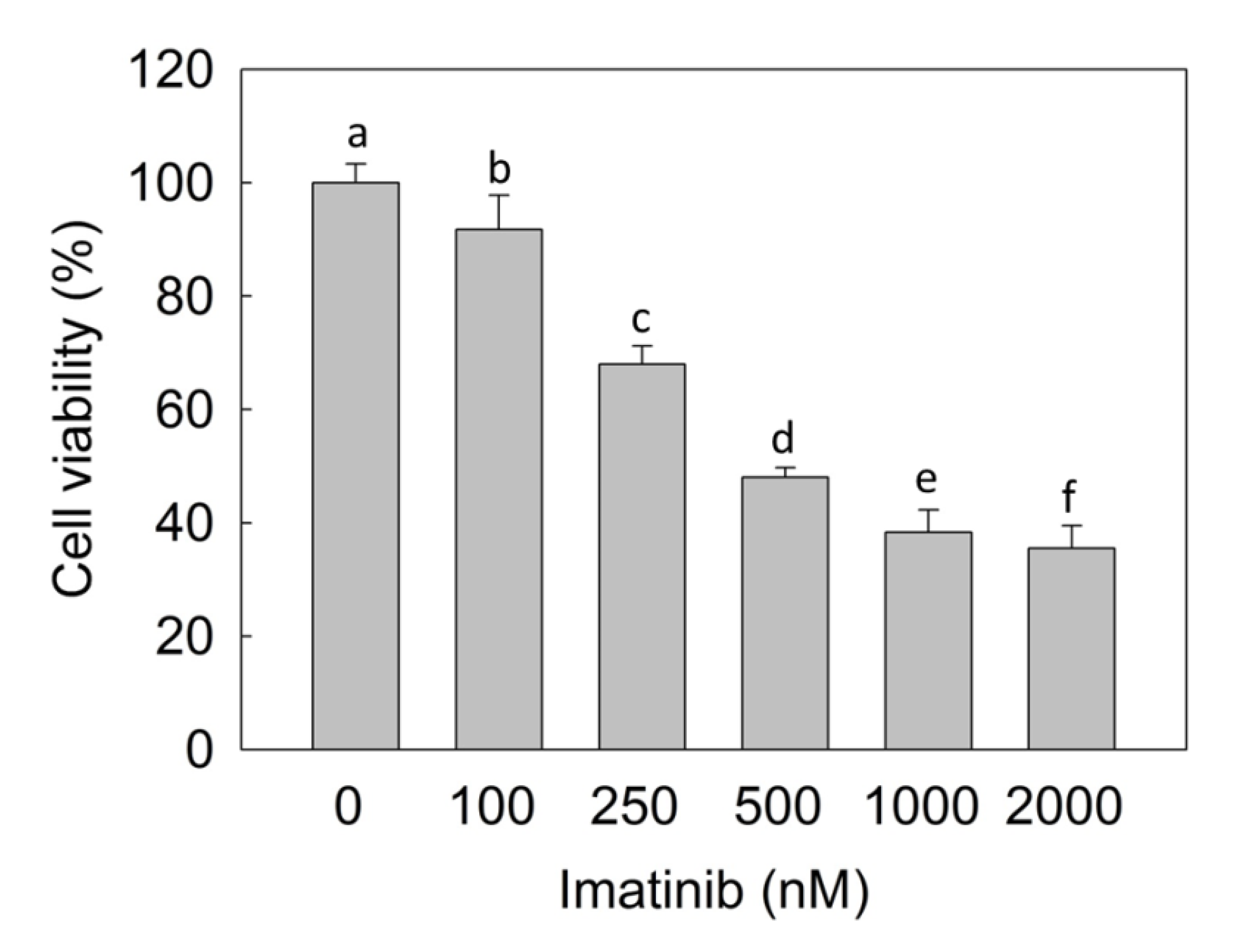

Supplement: S2 Fig — The viability of K562 cells was measured by an MTT assay after treatment with the indicated doses of IM for 48 h. The results are presented as means + SD of three independent experiments. Data abcdef without the same letter are significantly different from each group (p < 0.05). (TIFF) [file pone.0156260.s002.tiff]

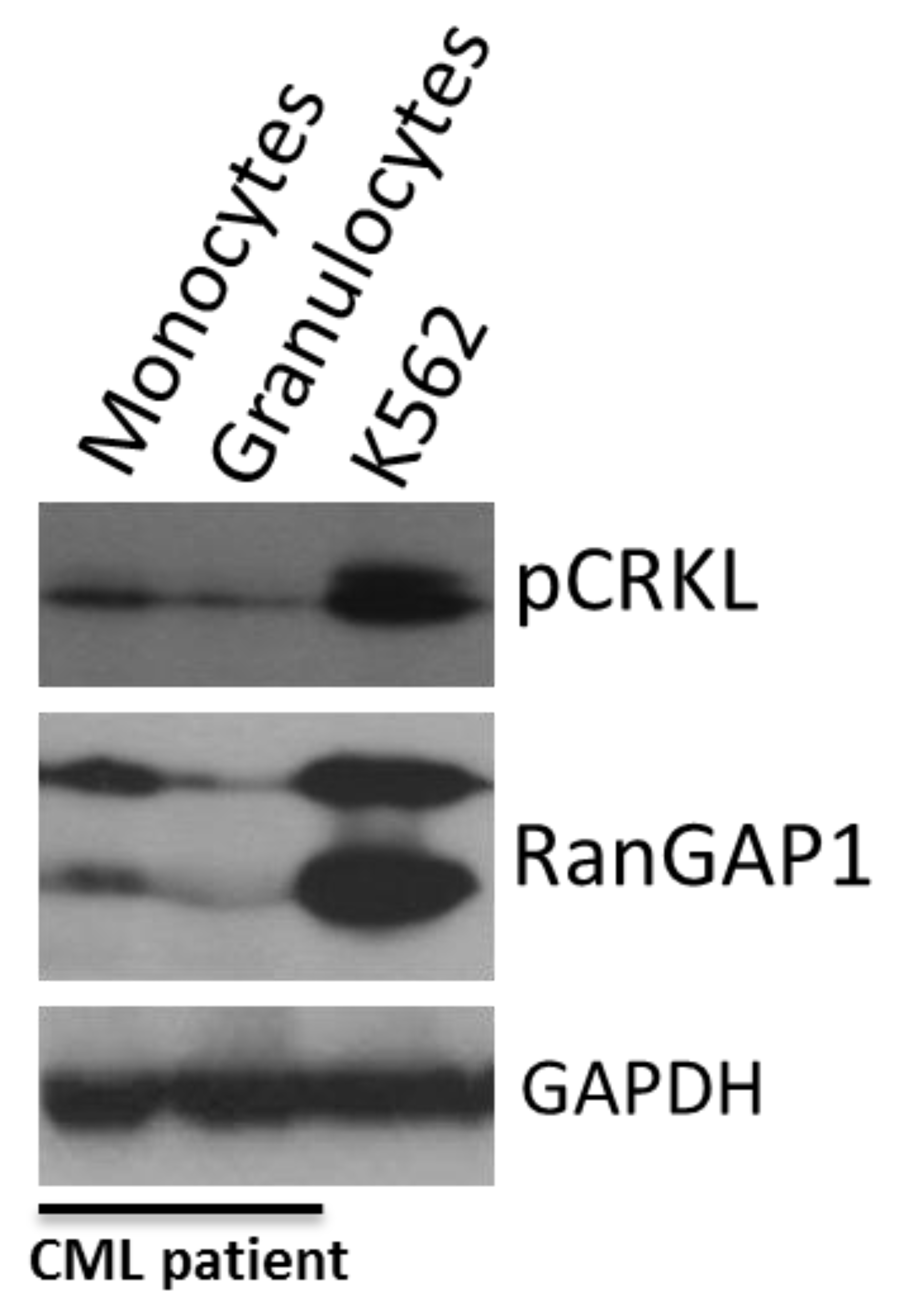

Supplement: S3 Fig — The RanGAP1 protein levels were measured using an immunoblot assay in granulocytes and monocytes from CML patient, and K562 cells. GAPDH was used as an internal control. The CRKL phosphorylation level on Tyr-207 is activated by BCR-ABL, which is used as a marker of CML cells. (TIFF) [file pone.0156260.s003.tiff]

## Slide 1
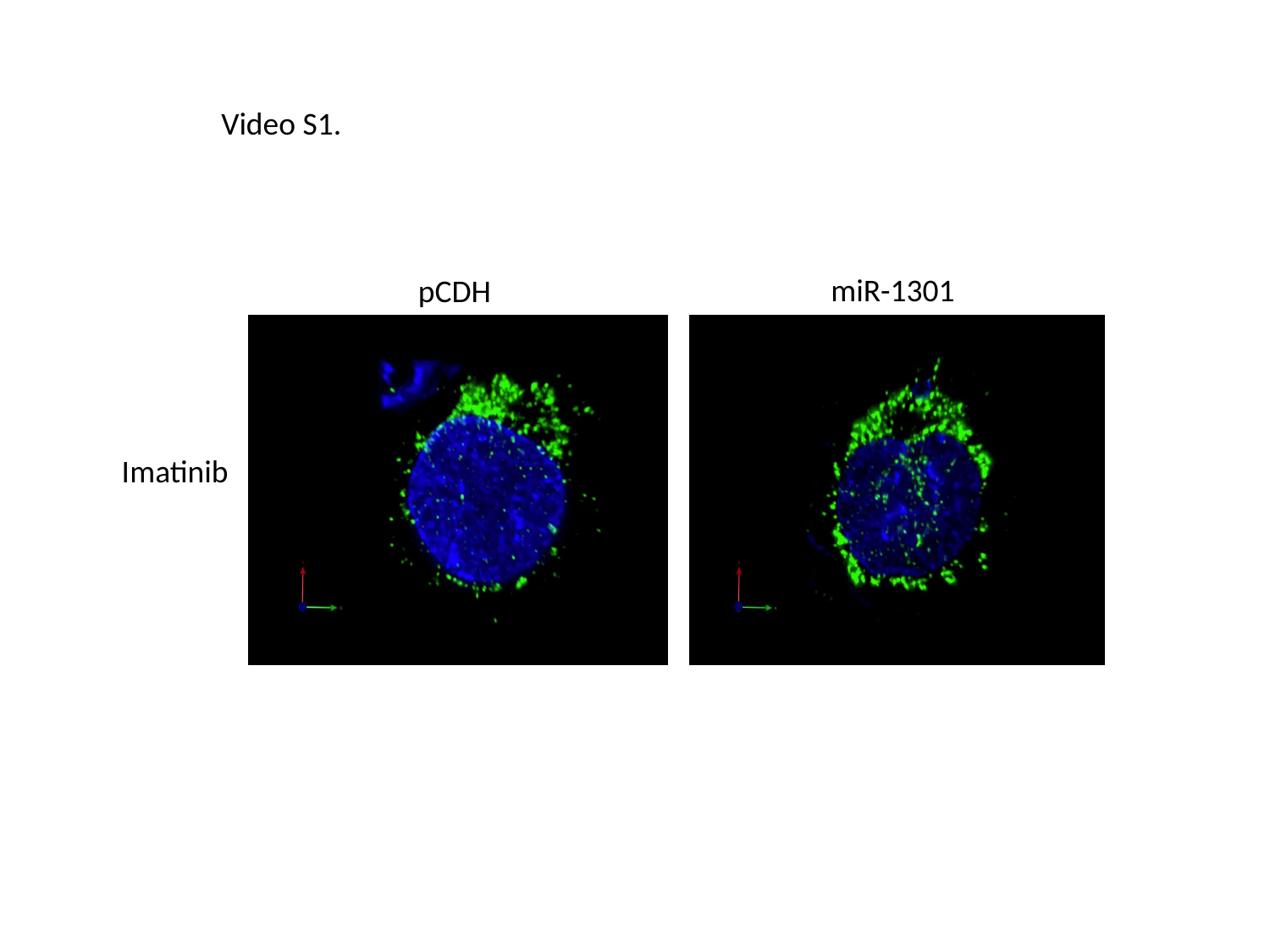

Video S1.
miR-1301
pCDH
Imatinib

Supplement: S1 File — K562 cells were transfected with pCDH (vector only) or the miR-1301 plasmid and subsequently treated with 250 nM IM for 48 h. The protein levels were observed using immunofluorescence staining through deconvolution microscopy as described in materials and methods. Video of various z-stack data from K562 cells expressing BCR-ABL (green) colabeled with the nuclear dye DAPI (blue). (PPTX) [file pone.0156260.s004.pptx]

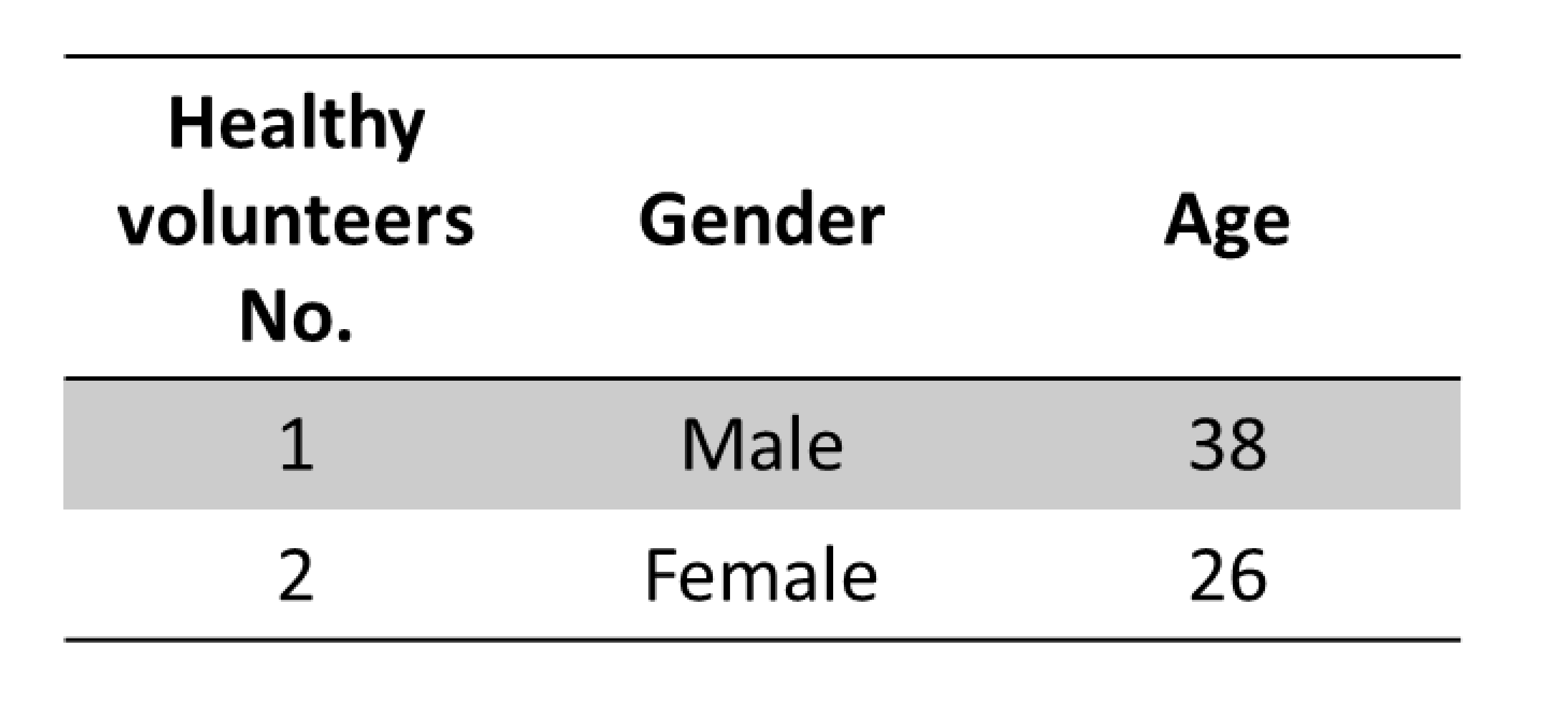

Supplement: S1 Table — (TIFF) [file pone.0156260.s005.tiff]

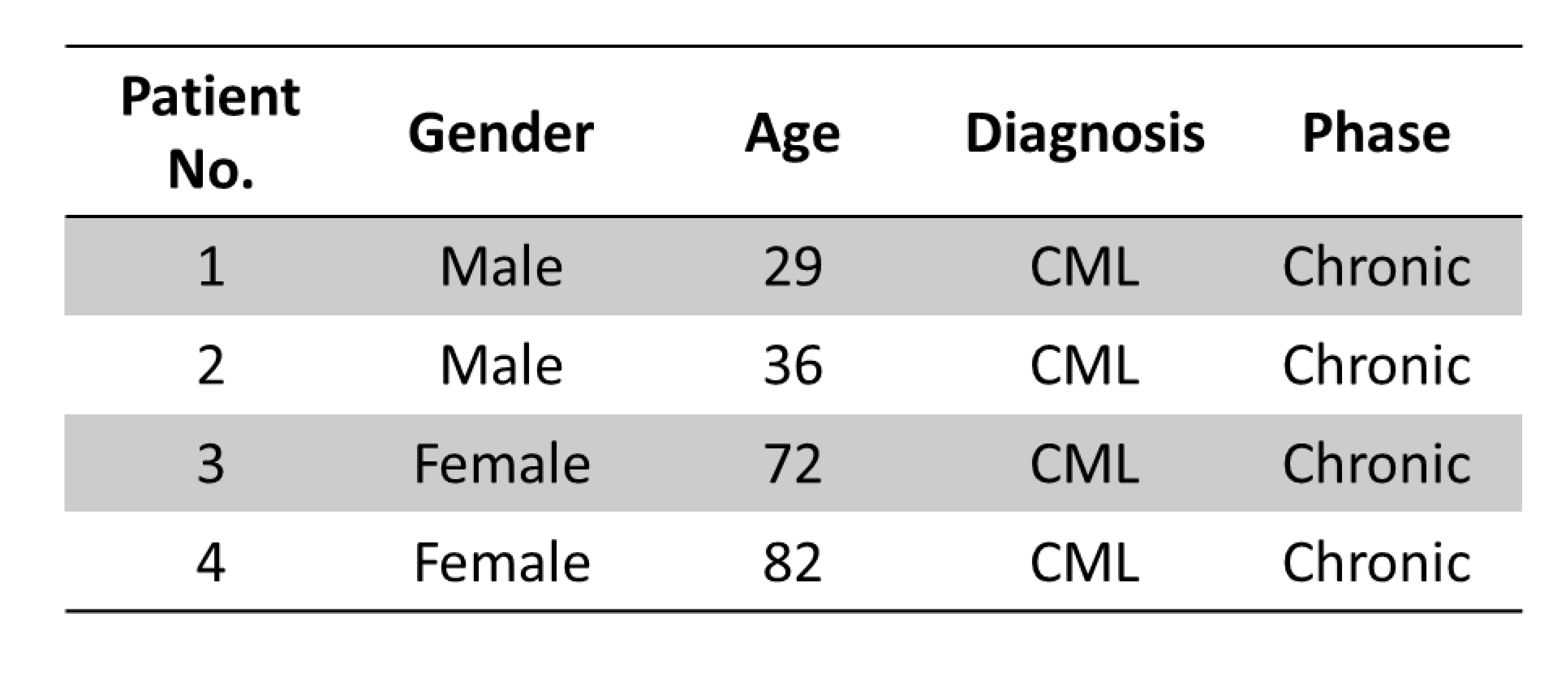

Supplement: S2 Table — (TIFF) [file pone.0156260.s006.tiff]
